# Birth Intervals and Associated Factors among Women Attending Young Child Clinic in Yumbe Hospital, Uganda

**DOI:** 10.1155/2020/1326596

**Published:** 2020-01-04

**Authors:** M. Aleni, S. N. Mbalinda, R. Muhindo

**Affiliations:** ^1^Department of Nursing and Midwifery, Muni University, +256 P.O. BOX, 725 Arua, Uganda; ^2^Department of Nursing, Makerere University, +256 P.O. BOX, 7062 Kampala, Uganda

## Abstract

**Introduction:**

Evidence suggests that both short and long birth intervals are associated with poor maternal and child health outcomes. However, current studies suggest that a number of births still occur at short intervals. The aim of this study was to document birth intervals and associated factors among women of reproductive age in rural Uganda.

**Materials and Methods:**

This was a cross-sectional study conducted among 296 women aged 15-49 years attending young child clinic at Yumbe Hospital who had at least two successive live births. Data was collected using interviewer-administered questionnaire. Birth interval was categorized according to the WHO-recommended birth interval of ≥24 months and <24 months.

**Results:**

Of the 296 participants, 86.6% desired a birth interval ≥ 24 months with a desired median birth interval of 36 months. The actual median birth interval was 22 months. Slightly more than half of the women (52.4%) had short birth intervals. Factors which were likely to be associated with short birth intervals included being younger (15-24 years) (AOR = 4.39, 95%CI = 1.49‐12.93, *P* = 0.007), not planning to have another pregnancy (AOR = 0.33, 95%CI = 0.18‐0.58, *P* = 0.001), not deciding together with husband when to have the next child (AOR = 3.10, 95%CI = 1.53‐6.28, *P* = 0.002), not always using contraceptives before the next pregnancy (AOR = 0.28, 95%CI = 0.12‐0.64, *P* = 0.003), and lack of influence of husband on when to have the next child (AOR = 2.59, 95%CI = 1.44–4.64, *P* = 0.001).

**Conclusion:**

Prevalence of short birth intervals is still high in rural Uganda (52.4%), although majority (86.6%) of the women desire optimal birth intervals. Factors which were likely to be associated with short birth intervals included young maternal age, not using contraceptives, and lack of male involvement in child spacing activities. Therefore, to optimize birth intervals, focused child spacing strategies targeting young women and men are needed.

## 1. Introduction

World Health Organization (WHO) defines birth interval as the time period from live birth to a successive pregnancy and the recommended period is at least 24 months (2 years) [1]. Short birth intervals defined as those less than 2 years have implications on maternal and child health [2], and long birth intervals defined as those above 5 years have undesired effects on maternal and child health as well [3, 4]. Adequate birth intervals on the other hand help women recover from macro- and micronutrient depletion which occurs during pregnancy and lactation [5]. This therefore helps to improve subsequent pregnancies and child health. It has been estimated that if all birth to pregnancy intervals were spaced at least 3 years, 1.6 million deaths of under-five would be averted annually [6].

Globally, it was estimated that 25% of births still occur at intervals less than 24 months. Most of the cases of short birth intervals were observed in Central Asia at 33%. Sub-Saharan Africa had short birth intervals at 20% [7]. Study of fertility transition in sub-Saharan Africa shows that the median birth interval at birth order two was 35 months which was longer than in Asia and Latin America of 25 months [8].

Though the sub-Saharan African picture shows optimal median birth intervals, individual countries were observed to be having differences in birth intervals. Demographic and Health Survey (DHS) reports show most of the countries having a significant number of births occurring at short intervals. Most countries in Africa including Uganda do not report about long birth intervals probably because it is not happening frequently. In Nigeria, short birth intervals were 23% [9], and in Zimbabwe, it was estimated at 11% [10]. In East Africa, Kenya and Tanzania had almost similar birth intervals less than 24 months of 18% [11] and 19% [12], respectively. Uganda Demographic and Health Survey (UDHS) 2016 did not report short birth, but [7] analysis of trends in birth intervals from DHS of several countries estimated that Uganda had short birth intervals occurring at a rate of 25.9%.

Studies have shown that short birth intervals contribute to poor maternal and child health [3, 13]. In Uganda, up to 4% of infant mortalities can be attributed to short birth intervals [14]. Some of the poor child health outcomes related to short birth intervals include preterm birth, low birth weight, congenital malformations, and early neonatal deaths [15]. Under-five mortality has also been found to increase with birth intervals below 2 years [16]. Among women, short birth interval is associated with maternal nutritional depletion, placenta previa and abruption, incomplete healing of caesarean section scars, poor lactation, and cross infection among siblings [5]. However, systematic review has shown that birth intervals longer than 5 years are associated with labour dystocia and increases the risk of preeclampsia [4]. Optimal birth intervals could therefore help prevent some of these poor maternal and child health outcomes.

Authors have documented a number of factors associated with birth intervals. Some of these may be direct factors such as being sexually active, use of contraceptives, postpartum infecundability, abortion, and sterility while indirect factors may include sociocultural factors [17]. In their study, Fayehun et al. [18] found that the type of contraceptives used, living in rural or urban settings, wealth index, husbands' occupation, and sex of preceding children determined birth intervals. It was further noted that having more female children and having a female last child significantly reduced birth intervals in quest for a male child. Use of contraceptives for birth spacing was also seen to be affected by son preference where the last child born being a female, a woman would like to use a shorter acting contraceptive in order not to wait too long to get a male child [19].

Uganda like many sub-Saharan African countries still has high fertility, maternal mortality, and neonatal, infant, and under-five mortality rates. Recent estimates show the countries total fertility rate at 5.4, maternal mortality rate at 336/100,000 live births, neonatal mortality rate at 27/1,000 live births, infant mortality rate at 43/1,000 live births, and under-five mortality rate at 64/1,000 live births [20]. However, little is known about the currents trends of birth intervals and its determinants. UDHS 2016 assessed preferred birth intervals for women of reproductive age associating it with only one factor (the number of living children a woman has). The actual birth intervals were not measured. Not many studies in Uganda have comprehensively studied contextual factors associated with birth intervals. Little is known about community awareness regarding dangers associated with short or long birth intervals.

Therefore, understanding birth intervals and its determinants is critical for a country like Uganda so as to improve and design child spacing programs which in turn will improve maternal and child health. This study therefore is aimed at filling this gap by assessing birth intervals and its determinants among women of reproductive age in rural Uganda.

## 2. Materials and Methods

### 2.1. Aim of the Study

The study was aimed at identifying birth intervals and its determinants among women of reproductive age in Yumbe district in rural Uganda.

### 2.2. Study Design

The study employed a cross-sectional design and quantitative methods for data collection.

### 2.3. Study Setting

The study was conducted in Yumbe Hospital at the maternal child health (MCH) unit, outpatient department. Yumbe Hospital is located in Yumbe district which is found in the West Nile subregion of Uganda, about 600 km from the capital Kampala. The hospital is located in a rural area in the southwestern part of the district about 10 kilometres away from Yumbe town. It is a grade V hospital, and services offered at the hospital include MCH, general medicine, surgery, and community health services. The hospital also serves as the district referral hospital. Mothers who come for MCH services are predominantly the indigenous people of the district, but a few clients are from the neighboring Koboko and Arua districts as well. Maternal child health services offered at the hospital at the outpatient department include antenatal care (ANC), postnatal care (PNC), young child clinic (YCC), and family planning services.

### 2.4. Study Population

The study participants were women aged 15-49 years who were attending YCC at Yumbe Hospital. A total of 296 women were selected using systematic sampling method. Women who had had two successive live births were included in the study. The study excluded women who had had two successive live births whose children were very sick and needed urgent treatment.

### 2.5. Sampling Procedure

Systematic sampling method was used. Systematic sampling is a probability sampling technique in which the study population is arranged according to some ordering scheme and then selecting elements at regular intervals. Only the first element is selected randomly, the rest being selected according to predetermined pattern. The predetermined pattern is *K*th unit which is the sampling interval (*K*) = *N*/*n*, where *N* = population size and *n* = sample size (Denise F pilot, 2004).

At the clinic, women and their children who were attending YCC were registered in the child health register. On data collection days, systematic sampling was achieved by sampling the *K*th woman in the register after the first one had been selected at random by blind folding one of the research assistants who was asked to put her index finger on any of the names which were recorded in the register on the first day of data collection. The count started with the name of the woman at the point where her index finger touched and the third person on the list being counted downwards from the random point was considered as the first participant, and the *K*th interval of 3 continued throughout the data collection period. If the *K*th woman did not meet the inclusion criteria, then the next woman on the list was selected and the interval continued. Once the list was completed for a data collection day, additional participants for the following day were sampled by looping the count to the beginning of the register for that day since women were being registered for the services on every working day at the clinic. This process was continued until all the required sample size was achieved. The selected participants were informed that the interview would be done as they exited the clinic so as not to interrupt their process of receiving health care.

### 2.6. Determining Sampling Interval (*K*)

*N* is the population sample size. *n* is the sample size. Time available for data collection was one month (20 working days). The average number of women received at YCC in a month was 1,020. Sampling interval (*K*) = 1,020/296 = 3.5. Therefore, the sampling interval was 3.

### 2.7. Study Variables

The outcome variable for this study was birth interval. Birth interval was measured in months using interval scale. Actual birth interval, desired birth interval, and proportion of nonfirst births that occurred before 24 months and after 24 months were measured. For actual and desired birth intervals, the last birth interval was measured.

#### 2.7.1. Actual Birth Interval

We defined actual birth interval as the time between a live birth and successive pregnancy. Only the last birth interval was measured. This was measured by assessing the length of time between the preceding birth and the current birth and then subtracting the length of pregnancy which resulted in the current birth, since women may not know their exact date of conception. Respondents were asked how old was your second last born child when your last baby (current baby) was born? They were then asked what was the duration of your pregnancy which resulted in the birth of the last (current) baby? Therefore, the difference between the age of the second last born child at the birth of the last born (current) child and the durations of pregnancy of the last born (current) child was considered as the actual birth interval. This was measured in months, e.g., if *a* is the age in months of the second last born child when the last (current) baby was born and if *b* is the duration of pregnancy which resulted in the birth of the last (current) baby, then actual birth interval (*c*) would be *a* − *b* = *c*. Multiple births were treated as a single interval. Therefore, birth interval was categorized according to the WHO-recommended birth interval (24 months) and coded 1 if birth interval was ≥24 months and 2 if birth interval was <24 months.

#### 2.7.2. Desired Birth Interval

This is the birth interval a woman would wish to have if all factors were favorable. This was measured in months by asking the following: at the time you became pregnant with the current baby, did you want to become pregnant then? If the woman said yes, then that was considered as her desired birth interval. If she said no, she was then asked the following: how long would you have preferred to wait from the time you gave birth to your second last born child to conception of your current baby? The length of time she mentioned was recorded as her desired birth interval.

#### 2.7.3. Proportion of Nonfirst Births That Occurred before 24 Months

This is the number of children a woman has excluding the first birth which occurred at an interval less than 24 months. This was measured by asking the following: how many of your children were born before 2 years of the preceding birth?

#### 2.7.4. Proportion of Nonfirst Births That Occurred after 24 Months

This is the number of children a woman has excluding the first birth which occurred at an interval more than 24 months. This was measured by asking the following: how many of your children were born after 2 years of the preceding birth?

### 2.8. Predictor Variables

In this study, independent variables are predisposing factors to birth intervals among women of reproductive age. This was measured by having precoded questions concerning factors that determine birth intervals ranked on an ordinal scale, and in some of the questions, the respondents were asked to choose response(s) of their choice.

#### 2.8.1. Demographic Characteristics

Demographic characteristics which were measured include age at first birth, current age of woman, education level, occupation, religion, marital status, residence, number of living children a woman has, and sex of the second last born child.

#### 2.8.2. Sociocultural Factors

Sociocultural factors were measured using an ordinal scale where the respondents were asked to indicate on a scale of 1 to 4 if she strongly agrees, agrees, disagrees, or strongly disagrees that the decision of her husband, mother-in-law, and significant others on when to have the next pregnancy matters a lot.

#### 2.8.3. Behavioral Factors

Behavioral factors that were measured include plan of when to have the next child, family planning use, and couple communication about birth intervals. Plan of when to have the next child and couple communication about birth interval were measured on an ordinal scale of 1 to 3 if she never, sometimes, and always discusses with her husband and decides when to have the next child or if she makes the decision alone. Family planning use was measured by asking the respondent if she never, sometimes, or always used contraceptives between pregnancies.

#### 2.8.4. Knowledge about Birth Intervals

Knowledge about birth intervals was measured by asking the respondents to mention the recommended birth interval. Respondents were also asked to choose precoded responses. Questions included awareness about the recommended birth intervals, dangers of short birth intervals, and the benefits of optimal birth intervals.

#### 2.8.5. Health Facility Factors

These are health facility-related factors that influence access and utilization of services that promote healthy birth intervals. Health system factors that were measured included the following: if health workers were giving information to the women about recommended birth intervals, contraceptive information, and provision of contraceptive services.

### 2.9. Data Collection Tools and Procedures

We used a questionnaire as a data collection tool. The questionnaire was formulated by the authors according to the objectives of the study using literature as a source. The questionnaire contained a total number of 37 structured questions and was formulated in English and translated in the local dialect (Aringa). The questionnaire included demographic characteristics which had 12 questions, questions related to birth intervals were 6 and factors which affect birth intervals were 19 in total (knowledge about birth intervals = 8, behavioral factors = 5, social factors = 3, and health facility factors = 3). Knowledge about birth intervals was measured by asking the respondents if they were aware about the recommended birth intervals and to mention the recommended birth interval, dangers of short birth intervals, and the benefits of optimal birth intervals. On average, the interview took approximately 20 minutes per participant. To ensure reliability of the questionnaire, questions were formulated according to the research objectives. The principal investigator pretested the questionnaire on 10 women attending YCC at Midigo HCIV in Yumbe district. Adjustments were made based on the results of the pretest by rephrasing questions which were not clear to the respondents and by removing questions which did not seem to be relevant. Reliability of the questionnaire was further ensured by selecting only respondents who met the inclusion. Furthermore, the research assistants were trained for half a day before data collection to ensure reliability of the data tool. All the filled questionnaires were checked every evening after a day's data collection by the principal investigator and the research assistants to ensure completeness and correctness of data collected. The respondents were identified from the child health register among mothers who were registered on data collection days. The principal investigator or the research assistant informed the participants of the purpose and objectives of the study, and consent was sought. The principal investigator or the research assistant read the questions on the questionnaire as the respondent listened. The respondents were required to select a precoded response. Responses were ticked or filled in accordingly by the principal investigator or research assistant.

### 2.10. Statistical Analysis

Data analysis was completed using SPSS version 23. At univariate analysis, descriptive statistics were summarized using mean (standard deviation), median (interquartile range), and range for continuous variables and frequencies and proportion for categorical variables (Tables 1–3). At bivariate analysis, chi-square test was used to assess the association between birth interval and factor variables. All the statistic tests and their respective confidence intervals (CI) were based on a two-tailed test (Table 4). At multivariate analysis, a logistic regression model to determine the effect of factor variables on birth interval was applied for simultaneous adjustment for potential confounders. Crude and adjusted odds ratio (OR) at 95% confidence interval and predictor variables with *P* value ≤ 0.1 at bivariate analysis and any other plausible variables were used to measure the association between dependent and predictive variables (Table 5). Variables with *P* value < 0.05 were considered statistically significant for the final statistical test. Data were presented using tables.

## 3. Results

The study was conducted in January 2018 among 296 women aged 15-49 years who had had at least two successive live births, attending young child clinic in rural Uganda.

### 3.1. Sociodemographic Characteristics of Respondents

The women in the study were aged between 18 and 43 years, with a mean age of 26.82 (SD: ±5.14). Their mean age at first birth was 17.34 (SD ± 2.39). Slightly more than half of the women (156, 52.7%) were aged 25-34 years and 112 (37.8%) were aged 15-24 years. Most of the women (279, 94.3%) lived in rural areas, and only 53 (17.9%) have attended secondary education or plus. Two hundred and thirty-five (79.4%) of the women were housewives. Majority (274, 92.6%) of the women were Muslims. Two hundred and eighty-three (96%) were married, and among them, 162 (57.2%) were in monogamous marriage. Majority of the women (235, 79.4%) had had between 2 and 4 live births. One hundred and twenty-seven (42.9%) of the women wanted their pregnancy at the time of conception. Nearly half of the participants (138, 46.6%) had never used any contraceptive method before their next pregnancy. One hundred and sixteen (39.2%) of the women always decide together with their husband on when to have the next child. One hundred and forty-one (48%) of the women acknowledged that the influence of their husbands matters a lot on when to have the next child, as shown in Table 1.

### 3.2. Awareness about Recommended Birth Intervals

As shown in Table 2, there was universal awareness about the recommended birth intervals. All the participants had heard about the recommended birth intervals, and all of them correctly mentioned the recommended birth intervals from 24 months and above. All the participants were able to mention at least one advantage of proper birth spacing and one disadvantage of short birth intervals. The major source of information about birth intervals was from health facilities.

### 3.3. Birth Intervals for Women of Reproductive Age

As shown in Table 3, using the WHO-recommended birth interval of ≥24 months, 52.4% of the women had birth intervals below 24 months. The median birth interval was 22 months (IQR = 15‐33) while the desired median birth interval was 36 months (IQR 36-36). Eighty-seven percent of the women desired birth intervals ≥ 24 months. Excluding the first birth for the 296 women studied, the participants had had 693 live births in total, and among them, 156 (22.5%) were born before two years of the preceding birth and 537 (77.5%) were born after two years of the preceding birth.

### 3.4. Bivariate Analysis

At bivariate analysis, chi-square test was used to explore the association between birth intervals and independent related variables.

As shown in Table 4, the unadjusted odds of short birth intervals were significantly higher among women aged 15-24 (COR = 5.67, 95% CI: 2.13-15.09, *P* = 0.001) and women aged 25-34 (COR = 3.96, 95% CI: 1.52-10.30, *P* = 0.005) compared to women aged 35-44. Other factors which were significantly associated with short birth intervals included not planning to have the next pregnancy, not deciding together with husband about when to have the next pregnancy, and not always using contraceptives before the next pregnancy. Women who planned to have their next pregnancy were 78% less likely to have short birth intervals (COR = 0.22, 95% CI: 0.13-0.35, *P* < 0.001) compared to those who did not plan to have the next pregnancy. Women who always decide together with their husbands when to have the next child were less likely to have short birth interval (COR = 2.85, 95% CI: 0.16-0.51, *P* < 0.001). The prevalence of short birth interval was 81% lower among women who always use contraceptive before the next pregnancy compared to those who never use contraceptives (COR = 0.19, 95% CI: 0.09-0.39, *P* < 0.001).

### 3.5. Multivariate Analysis

After bivariate logistic regression, variables with *P* value ≤ 0.1 and other plausible variables were further analyzed using multivariate logistic regression to control for confounding. The OR was adjusted for the following variables: maternal age, sex of the second last born child, whether the last pregnancy was wanted, contraceptive use, deciding together with husband when to have the next baby, and influence of husband on when to have the next baby. Variables with *P* ≤ 0.05 were considered significantly associated with birth intervals.

As shown in Table 5, factors associated with short birth intervals included young maternal age, not using contraceptives, not deciding together with husband when to have the next child, lack of husband influence on when to have the next baby, and not planning to have the next pregnancy. Women aged 15-24 years were 4 times more likely to have short birth intervals (AOR = 4.39, 95% CI: 1.49-12.93, *P* = 0.007), and those aged 25-34 years were 3 times more likely to have short birth intervals (AOR = 3.39, 95% CI: 1.18-9.74, *P* = 0.024) compared to women aged 35-44 years. The adjusted odds of short birth intervals were lower for women who wanted the pregnancy at the time of conception (AOR = 0.33, 95% CI: 0.18-0.58, *P* < 0.001), women who always used contraceptive before the next pregnancy (AOR = 0.28, 95% CI: 0.12-0.64, *P* = 0.003), those who always decide together with their husband on the time to have the next child (AOR = 3.10, 95% CI: 1.53-6.28, *P* = 0.002), and those who were influenced by their husband on when to have the next baby (AOR = 2.59, 95% CI: 1.44-4.64, *P* = 0.001).

## 4. Discussion

The aim of this study was to identify birth intervals and its determinants among women of reproductive age in rural Uganda. We measured the last birth interval for women who had had at least two live births. The study showed that slightly more than half of the study participants (52.4%) had short birth intervals. Many of the women in our study could not achieve their desired birth intervals probably because of low contraceptive prevalence where only 17.2% of the women always used contraceptives before the next pregnancy and 46.6% have never used a contraceptive method. Studies conducted in other parts of rural Uganda and Ethiopia reported similar results, where slightly more than half of the study population had short birth intervals [17, 21], unlike in Iran where only 3.8% of the women had short birth intervals [22]. Contrary to studies in Nigeria which posted higher prevalence of short birth intervals [23, 24], studies done in Uganda in general [7] and in Iran [25], Kenya [26], Bangladesh [27], and Tanzania [28] have posted lower prevalence of short birth intervals.

Median birth interval in our study was 22 months which is lower than the one recommended by WHO, yet majority of the women (86.6%) desired a birth interval of ≥24 months. These findings were consistent with studies done in Asian and Latin American countries where median birth intervals at birth order two was 25 months. Our median birth intervals were much lower than those in Ethiopia [29], in other sub-Saharan African countries [8], in South African countries [30], and in India [31]. Our study concurs with a study done in Saudi Arabia where a significantly lower proportion of the women (5.2%) desired short birth intervals [32].

Literature suggests a number of factors associated with birth intervals. In our study, the age of the woman, plan to have the next pregnancy, deciding together with husband when to have the next child, always using contraceptives before the next pregnancy, and influence of husband on when to have the next pregnancy were significantly associated with birth intervals.

Younger women were more likely to have short birth intervals compared to older women. This could probably be explained by the fact that fertility reduces with age above 35 years and older women could be having better ability to make independent decisions concerning their reproductive goals compared to the younger women. This may also be due to the fact that in this study, mean age at first birth was 17 years and older women might have been tending towards completing their family sizes. Our study findings are consistent with [22, 33], studies done in Democratic Republic of Congo and Bangladesh [27, 34], but contrast with studies done in Iraq where being younger was associated with longer birth intervals [25].

Women who planned their pregnancy were less likely to have short birth intervals compared to those who did not want the pregnancy at the time of conception. This could probably be due to the fact that women/couples who plan their pregnancy may follow the recommendations for child spacing and therefore end up with optimal birth intervals. Limited access to reproductive health services in some settings may lead to unwanted or unplanned pregnancies for those women who wish to use contraceptives at present or in the future, leading to the notion of unmet need for family planning and therefore short birth intervals [35]. Our study is consistent with a study done in the Philippines where women who had a final say about their desired birth intervals had longer birth intervals [36].

Our study found that women who always decide together with their husband on when to have the next child were less likely to have short birth intervals. This could probably be argued that proper communication between couples about reproductive goals has a positive outcome on birth spacing. The level of reproductive health decision-making among couples such as when to have the next pregnancy, family size, and family planning use among others can result in benefits which impact positively on the family in general [37]. These results are consistent with a study done in ten sub-Saharan African countries where couples who agreed about when to have the next pregnancy had significantly longer birth intervals [38].

Women whose husbands have influence on when to have the next baby were less likely to have short birth intervals. This could probably be argued that these husbands supported interventions such as contraceptive use and other interventions which promote adequate birth intervals. These findings are consistent with studies done in rural Uganda, Saudi Arabia, and Egypt [21, 32, 39] where the lack of partner support was found to be associated with short birth intervals.

Lack of contraceptive use has been documented in literature as one of the strongest predictors of short birth intervals [29]. Contraceptives delay conception after child birth and are one of the best practices that can lead to achievement of recommended birth intervals and therefore optimal maternal and child health outcomes [40]. As expected, this was demonstrated in our study. Only 17.2% of the women in our study always used contraceptives before the next pregnancy, hence the high proportion of short birth intervals. Our study is consistent with studies done in Northern Iran, Bangladesh, and Nigeria which reported significant association between contraceptive use and optimal birth intervals [18, 27, 41] but contrasts with a study done in Democratic Republic of Congo which did not show any significant association between modern contraceptive use and child spacing [34].

There was a universal awareness about recommended birth intervals in our study where all the respondents correctly mentioned the WHO-recommended birth intervals. All the respondents were able to mention at least one advantage of optimal birth intervals and one disadvantage of short birth intervals, but this knowledge was not translated into practice of optimal birth intervals by the majority of the respondents, where slightly more than half of the respondents had short birth intervals. This could probably be argued that factors other than knowledge might have resulted in this high proportion of short birth intervals. Similar results were found in Egypt where there was no significant difference in birth intervals with the control group even though the experimental group was given information about the recommended birth interval and advantages of healthy birth spacing [42].

Although literature shows that mothers' education level [21], formal employment [43], sex preference [23], and number of living children [23, 27] are associated with birth intervals, this was not demonstrated in our study. The absence of association between education level, formal employment, and birth intervals could probably be explained by less variability in the study population with only 18% of the respondents having reached secondary school level or above and only 6.8% of the study population being formally employed.

The following limitations should be considered when interpreting the results of this study. The source of data for this study was based on the self-report of mothers, and no validation of information was made with any objective sources such as health facility cards except for immunization card of the current baby. This could have introduced recall bias which was controlled by only asking the last birth interval. Respondents were also critically informed about the importance of giving accurate information by assuring the confidentiality of their responses, and it is logical to assume that biases are less likely in birth interval events than other more sensitive events. Finally, the sample size of this study may affect generalization of the results of factors to the whole population. However, a study involving a bigger sample size is necessary.

## 5. Conclusion

In this study, we observed relatively high prevalence of short birth intervals among rural women in Uganda despite adequate knowledge about the recommended birth intervals and the majority desiring optimal birth intervals. Factors which were likely to influence short birth intervals included younger maternal age, not using contraceptives, not planning for pregnancies, not deciding together with their husbands about when to have the next child, and lack of influence of husband on when to have the next baby. We recommend that other than imparting knowledge on recommended birth interval, Ugandan ministry of health together with other stakeholders should strengthen the existing strategies of family planning as a comprehensive package in order to promote optimal birth intervals. Specific programs should be designed to deliberately target young women and men with family planning services. A study assessing how best men can be reached with reproductive health services could be conducted in order to improve their involvement in child spacing.

## Figures and Tables

**Table 1:** Distribution of background characteristics of women aged 15-49 years attending YCC at Yumbe Hospital, Uganda.

Variable	Frequency (*N* = 296)	Percentage	Mean
Age			
15-24	112	37.8	
25-34	156	52.7	26.82 (SD ± 5.14)
35-44	28	9.5	
Age at first birth			17.34 (SD ± 2.39)
Residence			
Rural	279	94.3	
Urban	17	5.7	
Marital status			
Married	283	95.6	
Single & divorced	13	4.4	
Marriage status (283)^∗^			
Polygamous	121	42.8	
Monogamous	162	57.2	
Education level			
No education	57	19.3	
Primary	186	62.8	
Secondary+	53	17.9	
Religion			
Muslim	274	92.6	
Non-Muslim	22	7.4	
Occupation			
Housewife	235	79.4	
Formal employment	20	6.8	
Informal employment	41	13.9	
Number of live births			
5+	61	20.6	
2-4	235	79.4	
Sex of the 2^nd^ last born			
Female	147	49.7	
Male	149	50.3	
Pregnancy wanted			
No	169	57.1	
Yes	127	42.9	
Contraceptive use before the next pregnancy			
Never	138	46.6	
Sometimes	107	36.1	
Always	51	17.2	
Decide together with husband when to have next child			
Never	93	31.4	
Sometimes	87	29.4	
Always	116	39.2	
Influence of husband when to have the next baby			
No	155	52.4	
Yes	141	47.6	

^∗^13 of the respondents were not married.

**Table 2:** Awareness about recommended birth intervals among women attending YCC in rural Uganda (*n* = 296).

Variable	Frequency	Percentage	Mean
Heard about recommended birth interval			
Yes	296	100	
No			
Knowledge on recommended birth intervals			
≥24 months	296	100	37.77 (SD ± 10.24)
<24 months			
Source of information			
Health facility	266	98.90	
Peers	195	65.90	
Family	136	45.90	
Radio	103	34.80	
Benefits of optimal birth intervals (≥24 months)			
Good nutrition for family	251	84.80	
Adequate time for care of children	282	95.30	
Adequate lactation	77	26.00	
Adequate time for income generation activities	213	72.00	
Good health of the mother	10	03.20	
Dangers of short birth intervals (<24 months)			
Premature birth	91	30.70	
Low birth weight	165	55.70	
Poor lactation	137	46.30	
Death of the baby	202	68.20	
Death of the mother	136	45.90	
Poor health of the child	66	22.30	

^∗^Multiple responses.

**Table 3:** Birth intervals among women attending YCC at Yumbe Hospital, Uganda.

Variable	Frequency (*N* = 296)	Percentage	Median	Mean
Actual birth interval				
<24 months	155	52.4	22 (IQR: 18)	25.94 (SD ± 14.8)
≥24 months	141	47.6		
Desired birth intervals				
<24 months	40	13.3	36 (IQR: 0)	37.77 (SD ± 10.24)
≥24 months	256	86.6		
Children born before two years (*n* = 693)				
Yes	156	22.5		
No	537	77.5		

**Table 4:** Bivariate analysis of factors and short birth intervals among women attending YCC in rural Uganda.

Factors	Number (and %) of women with the birth interval		COR (95% CI)	*P* value
	<24 months *N* = 155 (52.4)	≥24 months *N* = 141 (47.6)		
Age				
35-44	6 (21.4)	22 (78.6)	1.0	
25-34	81 (51.9)	75 (48.1)	3.96 (1.52-10.30)	0.005
15-24	68 (60.7)	44 (39.3)	5.67 (2.13-15.09)	0.001
Residence				
Rural	146 (52.3)	133 (47.7)	1.0	
Urban	9 (52.9)	8 (47.1)	1.02 (0.38-2.73)	0.961
Marital status				
Married	150 (53.0)	133 (47.0)	1.0	
Single & divorced	5 (38.5)	8 (61.5)	0.55 (0.18-1.74)	0.311
Education level				
No education	26 (45.6)	31 (54.4)	1.0	
Primary	104 (55.9)	82 (44.1)	1.51 (0.83-2.74)	0.174
Secondary+	25 (47.2)	28 (52.8)	1.06 (0.50-2.25)	0.870
Religion				
Muslim	145 (52.9)	129 (47.1)	1.0	
Non-Muslim	10 (45.5)	12 (54.5)	0.74 (0.31-1.77)	0.501
Occupation				
Housewife	130 (55.3)	105 (44.7)	1.0	
Formal employment	8 (40.0)	12 (60.0)	0.54 (0.21-1.37)	0.192
Informal employment	17 (41.5)	24 (58.5)	0.57 (0.29-1.12)	0.104
Number of live births				
5+	29 (47.5)	32 (52.5)	1.0	
2-4	126 (53.6)	109 (46.4)	1.28 (0.73-2.24)	0.398
Sex of the 2^nd^ last born				
Female	68 (46.3)	79 (53.7)	1.0	
Male	87 (58.4)	62 (41.6)	1.63 (1.03-2.58)	0.037
Pregnancy wanted				
No	115 (68.0)	54 (32.0)	1.0	
Yes	40 (31.5)	87 (68.5)	0.22 (0.13-0.35)	<0.001
Contraceptive use before the next pregnancy				
Never	86 (62.3)	52 (37.7)	1.0	
Sometimes	57 (53.3)	50 (46.7)	0.69 (0.41-1.15)	
Always	12 (23.5)	39 (76.5)	0.19 (0.09-0.39)	<0.001
Decide together with husband when to have next child				
Never	54 (36.7)	28 (21.2)	1.0	
Sometimes	53 (36.1)	34 (24.8)	4.00 (0.45-1.50)	<0.001
Always	40 (27.2)	74 (54.0)	2.85 (0.16-0.51)	<0.001
Influence of husband when to have the next baby				
No	86 (58.1)	62 (41.9)	1.0	
Yes	60 (46.1)	74 (53.9)	1.70 (0.39-098)	0.033

**Table 5:** Predictors of short birth intervals among study respondents.

Variable	Number (and %) of women with the birth interval		Unadjusted OR (95% CI)	*P* value	AOR (95% CI)	*P* value
	<24 months *N* = 155 (52.36)	≥24 months *N* = 141 (47.64)				
Age						
35-44	6 (21.4)	22 (78.6)	1.0		1.0	
25-34	81 (51.9)	75 (48.1)	3.96 (1.52-10.30)	0.005	3.39 (1.18-9.74)	0.024
15-24	68 (60.7)	44 (39.3)	5.67 (2.13-15.09)	0.001	4.39 (1.49-12.93)	0.007
Sex of second last born child						
Female	68 (46.3)	79 (53.7)	1		1	
Male	87 (58.4)	62 (41.6)	0.54 (0.32–0.93)	0.027	0.61 (0.35–1.05)	0.077
Pregnancy wanted						
No	115 (68.0)	54 (32.0)	1.0		1.0	
Yes	40 (31.5)	87 (68.5)	0.22 (0.13-0.35)	<0.001	0.33 (0.18-0.58)	<0.001
Decide together with husband when to have child						
Never	54 (36.7)	28 (21.2)	1.0		1.0	
Sometimes	53 (36.1)	34 (24.8)	4.43 (2.16–9.07)	<0.001	2.84 (1.32–6.11)	<0.008
Always	40 (27.2)	74 (54.0)	3.75 (1.88–7.47)	<0.001	3.10 (1.53–6.28)	0.002
Contraceptive use before next pregnancy						
Never	86 (62.3)	52 (37.7)	1.0		1.0	
Sometimes	57 (53.3)	50 (46.7)	0.69 (0.41-1.15)	0.155	0.59 (0.32-1.08)	0.085
Always	12 (23.5)	39 (76.5)	0.19 (0.09-0.39)	<0.001	0.28 (0.12-0.64)	0.003
Influence of husband on when to the next baby						
No	86 (58.1)	62 (41.9)	1.0		1.0	
Yes	60 (46.1)	74 (53.9)	2.68 (1.51–4.76)	0.001	2.59 (1.44–4.64)	0.001

## Data Availability

The primary data used to support the findings of this study are available from the corresponding author upon request.
